# Supplementary material for: Cost-Effective Method for Full-Length Sequencing of Monoclonal Antibodies from Hybridoma Cells
Source: Antibodies (Basel). 2025 Aug 22;14(3):72. doi: 10.3390/antib14030072 (PMC12452578; doi:10.3390/antib14030072)
Supplement: Supplementary file 1 [file antibodies-14-00072-s001.zip › Supplementary Material_2025-08-15.pdf]

antibodies

MDPI

Supplementary materials

Cost-effective method for full-length sequencing of monoclonal antibodies from hybridoma cells

Sarah Döring, Georg Tscheuschner, Sabine Flemig, Michael G. Weller and Zoltán Konthur*

Federal Institute for Materials Research and Testing (BAM), Richard-Willstätter-Strasse 11, 12489 Berlin, Germany
*Correspondence: zoltan.konthur@bam.de, Tel.: +49-30-8104-5840

Note: All protein and nucleotide sequences of reference sequences and antibody sequence of clone BAM-CCMV-29-81 are available as machine-readable text files in the Supplement Material.

Table S1: Overview of all used primers in this paper.

Name	Sequence (5′ to 3′)	Reference
template-switch oligo	AAGCAGTGGTATCAACGCAGAGTACATrGrGrG	
mIGK RT	TTGTCGTTCACTGCCATCAATC	
mIGL RT	GGGGTACCATCTACCTTCCAG	
mIGHG RT	AGCTGGGAAGGTGTGCACAC	[21]
ISPCR	AAGCAGTGGTATCAACGCAGAG	
mIGK PCR	ACATTGATGTCTTTGGGGTAGAAG	
mIGL PCR	ATCGTACACACCAGTGTGGC	
mIGHG PCR	GGGATCCAGAGTTCCAGGTC	
AOligo(dT)-ISPCR	AAGCAGTGGTATCAACGCAGAGTTTTTTTTTTTTTTTTTTTTVN	
CCMV-mIGHG_VDJ-PCR	TCAGATACGGTGGTGGAGGGTT	This study
CCMV-mIGK_VJ-PCR	GTTACCCACGGACGTTCGGTGGA	
pJET1.2 Forward	CGACTCACTATAGGGAGAGCGGC	Thermo-Fisher
pJET1.2 Reverse	AAGAACATCGATTTTCCATGGCAG	

Table S2: Overview of functional constant heavy chain mouse DNA reference sequences used for data analysis. Strains: C57BL/6J[1], BALB/c[2] and NOD[3]. *CH3 contain also the secreted membrane DNA sequence CHS.

Gene	NCBI Acc. Nr.	Region	DNA Sequence
IGHG1	NC_000078.7[1]	CH1	CCAAAACGACACCCCCATCTGTCTATCCACTGGCCCCTGGATCTGCTGCCCAAACTAAC TCCATGGTGACCCTGGGATGCCTGGTCAAGGGCTATTTCCCTGAGCCAGTGACAGTGAC CTGGAACTCTGGATCCCTGTCCAGCGGTGTGCACACCTTCCCAGCTGTCCTGCAGTCTG ACCTCTACACTCTGAGCAGCTCAGTGACTGTCCCCTCCAGCACCTGGCCCAGCCAGAC CGTCACCTGCAACGTTGCCCACCCGGCCAGCAGCACCAAGGTGGACAAGAAAATTG
IGHG2A	NT_096355.1[2]	CH1	CCAAAACAACAGCCCCATCGGTCTATCCACTGGCCCCTGTGTGTGGAGATACAACTGG CTCCTCGGTGACTCTAGGATGCCTGGTCAAGGGTTATTTCCCTGAGCCAGTGACCTTGA CCTGGAACTCTGGATCCCTGTCCAGTGGTGTGCACACCTTCCCAGCTGTCCTGCAGTCT GACCTCTACACCCTCAGCAGCTCAGTGACTGTAACCTCGAGCACCTGGCCCAGCCAGT CCATCACCTGCAATGTGGCCCACCCGGCAAGCAGCACCAAGGTGGACAAGAAAATTG
IGHG2B	NC_000078.7[1]	CH1	CCAAAACAACACCCCCATCAGTCTATCCACTGGCCCCTGGGTGTGGAGATACAACTGG TTCCTCTGTGACTCTGGGATGCCTGGTCAAGGGCTACTTCCCTGAGTCAGTGACTGTGA CTTGGAACTCTGGATCCCTGTCCAGCAGTGTGCACACCTTCCCAGCTCTCCTGCAGTCT GGACTCTACACTATGAGCAGCTCAGTGACTGTCCCCTCCAGCACCTGGCCAAGTCAGA CCGTCACCTGCAGCGTTGCTCACCCAGCCAGCAGCACCACGGTGGACAAAAAACTTG
IGHG2C	NC_000078.7[1]	CH1	CCAAAACAACAGCCCCATCGGTCTATCCACTGGCCCCTGTGTGTGGAGGTACAACTGG CTCCTCGGTGACTCTAGGATGCCTGGTCAAGGGTTATTTCCCTGAGCCAGTGACCTTGA CCTGGAACTCTGGATCCCTGTCCAGTGGTGTGCACACCTTCCCAGCTCTCCTGCAGTCT GGCCTCTACACCCTCAGCAGCTCAGTGACTGTAACCTCGAACACCTGGCCCAGCCAGA CCATCACCTGCAATGTGGCCCACCCGGCAAGCAGCACCAAAGTGGACAAGAAAATTG
IGHG2C	NC_000078.7[1]	Hinge	AGCCCAGAGTGCCCATAACACAGAACCCCTGTCCTCCACTCAAAGAGTGTCCCCCATG CGCAG
IGHG2C	NC_000078.7[1]	CH2	CTCCAGACCTCTTGGGTGGACCATCCGTCTTCATCTTCCCTCCAAAGATCAAGGATGTA CTCATGATCTCCCTGAGCCCCATGGTCACATGTGTGGTGGTGGATGTGAGCGAGGATGA CCCAGACGTCCAGATCAGCTGGTTTGTGAACAACGTGGAAGTACACACAGCTCAGACA CAAACCCATAGAGAGGATTACAACAGTACTCTCCGGGTGGTCAGTGCCCTCCCCATCC AGCACCAGGACTGGATGAGTGGCAAGGAGTTCAAATGCAAGGTCAACAACAGAGCCC TCCCATCCCCCATCGAGAAAACCATCTCAAAACCCAGAG
IGHG2C	NC_000078.7[1]	CH3-CHS*	GGCCAGTAAGAGCTCCACAGGTATATGTCTTGCCTCCACCAGCAGAAGAGATGACTAA GAAAGAGTTCAGTCTGACCTGCATGATCACAGGCTTCTTACCTGCCGAAATTGCTGTGG ACTGGACCAGCAATGGGCGTACAGAGCAAAACTACAAGAACACCGCAACAGTCCTGG ACTCTGATGGTTCTTACTTCATGTACAGCAAGCTCAGAGTACAAAAGAGCACTTGGGA AAGAGGAAGTCTTTTCGCCTGCTCAGTGGTCCACGAGGTGCTGCACAATCACCTTACGA CTAAGACCATCTCCCGGTCTCTGGGTAAA
IGHG2C	Y10606.1[3]	CH3-CHS*	GGCCAGTAAGAGCTCCACAGGTATATGTCTTGCCTCCACCAGCAGAAGAGATGACTAA GAAAGAGTTCAGTCTGACCTGCATGATCACAGGCTTCTTACCTGCCGAAATTGCTGTGG ACTGGACCAGCAATGGGCGTACAGAGCAAAACTACAAGAACACCGCAACAGTCCTGG ACTCTGATGGTTCTTACTTCATGTACAGCAAGCTCAGAGTACAAAAGAGCACTTGGGA AAGAGGAAGTCTTTTCGCCTGCTCAGTGGTCCACGAGGGTCTGCACAATCACCTTACGA CTAAGACCATCTCCCGGTCTCTGGGTAAA
IGHG3	NC_000078.7[1]	CH1	CTACAACAACAGCCCCATCTGTCTATCCCTTGGTCCCTGGCTGCGGTGACACATCTGGA TCCTCGGTGACACTGGGATGCCTTGTCAAAGGCTACTTCCCTGAGCCGGTAACTGTAAA ATGGAACTATGGAGCCCTGTCCAGCGGTGTGCGCACAGTCTCATCTGTACTGCAGTCTG GGTTCTATTCCCTCAGCAGCTTGGTGACTGTACCCTCCAGCACCTGGCCCAGCCAGACT GTCATCTGCAACGTAGCCCACCCAGCCAGCAAGACTGAGTTGATCAAGAGAATCG

Table S3: Overview of functional constant light chain mouse DNA reference sequences used for data analysis. Strain: C57BL/6J[1].

Gene	NCBI Acc. Nr.	DNA Sequence
IGKC	NC_000072.7[1]	GGGCTGATGCTGCACCAACTGTATCCATCTTCCCACCATCCAGTGAGCAGTTAACATCTGGAGGT<br>GCCTCAGTCGTGTGCTTCTTGAACAACTTCTACCCCAAAGACATCAATGTCAAGTGGAAGATTGA<br>TGGCAGTGAACGACAAAATGGCGTCCTGAACAGTTGGACTGATCAGGACAGCAAAGACAGCAC<br>CTACAGCATGAGCAGCACCCTCACGTTGACCAAGGACGAGTATGAACGACATAACAGCTATACC<br>TGTGAGGCCACTCACAAGACATCAACTTCACCCATTGTCAAGAGCTTCAACAGGAATGAGTGT
IGLC1	NC_000082.7[1]	GCCAGCCCAAGTCTTCGCCATCAGTCACCCTGTTTCCACCTTCCTCTGAAGAGCTCGAGACTAACA<br>AGGCCACACTGGTGTGTACGATCACTGATTTCTACCCAGGTGTGGTGACAGTGGACTGGAAGGTA<br>GATGGTACCCCTGTCACTCAGGGTATGGAGACAACCCAGCCTTCCAAACAGAGCAACAACAAGT<br>ACATGGCTAGCAGCTACCTGACCCTGACAGCAAGAGCATGGGAAAGGCATAGCAGTTACAGCTG<br>CCAGGTCACTCATGAAGGTCACACTGTGGAGAAGAGTTTGTCCCGTGCTGACTGTTCCTAG
IGLC2	NC_000082.7[1]	GTCAGCCCAAGTCCACTCCCACTCTCACCGTGTTTCCACCTTCCTCTGAGGAGCTCAAGGAAAAC<br>AAAGCCACACTGGTGTGTCTGATTTCCAACTTTTCCCCGAGTGGTGTGACAGTGGCCTGGAAGGC<br>AAATGGTACACCTATCACCCAGGGTGTGGACACTTCAAATCCCACCAAAGAGGGCAACAAGTTC<br>ATGGCCAGCAGCTTCCTACATTTGACATCGGACCAGTGGAGATCTCACAACAGTTTTACCTGTCA<br>AGTTACACATGAAGGGGACACTGTGGAGAAGAGTCTGTCTCCTGCAGAATGTCTCTAA
IGLC3	NC_000082.7[1]	GTCAGCCCAAGTCCACTCCCACACTCACCATGTTTCCACCTTCCCCTGAGGAGCTCCAGGAAAAC<br>AAAGCCACACTCGTGTGTCTGATTTCCAATTTTTCCCCAAGTGGTGTGACAGTGGCCTGGAAGGC<br>AAATGGTACACCTATCACCCAGGGTGTGGACACTTCAAATCCCACCAAAGAGGACAACAAGTAC<br>ATGGCCAGCAGCTTCTTACATTTGACATCGGACCAGTGGAGATCTCACAACAGTTTTACCTGCCA<br>AGTTACACATGAAGGGGACACTGTGGAGAAGAGTCTGTCTCCTGCAGAATGTCTC
IGLC4	NC_000082.7[1]	GCCAACCCAAGGCTACACCCTCAGTTAATCTGTTCCCACCTTCCTCTGAAGAGCTCAAGACTAAA<br>AAGGCCACACTGGTGTGTATGATCACTGAGTTCTACGCAGCTGCTGTGAGAGTGGCCTGGAAGGC<br>AGATGGTACCCCTTTCACTCAGGGTGTAGAGACTACCCAGCCTCCCAAACAGAGGGACAACATG<br>GCTAGCAGTTACCTGCTCTTCACAGCAGAAGCGTGGGAATCTCATAGCAGTTACAGCTGCCATGT<br>CACTCATGAAGGGCAACATGTGGAGAAGAGTTTGTCCCGTGCTGAGTGTTCC

Table S4: Overview of amino acid sequence from mouse monoclonal antibody of clone BAM-CCMV-29-81. Leader sequence is underlined.

Chain	Amino acid Sequence
Kappa-chain of<br>Anti-CCMV-Antibody<br>(BAM-CCMV-29-81)	MDFQVQIFSFLLISASVIMSRGQIVLTQSPAIMSASPGEKVTISCSASSSISYMYWYQQKPGSSPKSWIYRTSNLASGV<br>PARFSGSGSGTSYSLTISSMEAEDAATYYCQQYHSYPRTFGGGTKLEIKRADAAPTVSIFPPSSEQLTSGGASVVCFL<br>NNFYPKDINVKWKIDGSERQNGVLNSWTDQDSKDSTYSMSSTLTLTKDEYERHNSYTCEATHKTSTSPIVKSFNR<br>NEC
Heavy-chain IgG2c of<br>Anti-CCMV-Antibody<br>(BAM-CCMV-29-81)	MAVLALLLCLVTFPSCILSQVQLKESGPGLVAPSQSLSITCTVSGFSLSSYVVDWVRQSPGKGLEWLGVIWGVGSTN<br>YNSALKSRLSISKDNSKSQVFLKMNSLQTDDTAMYYCVRYGGGGFAYWGQGTLVTVSAAKTTAPSVYPLAPVCG<br>GTTGSSVTLGCLVKGYFPEPVTLTWNSGSLSSGVHTFPALLQSGLYTLSSSVTVTSNTWPSQTITCNVAHPASSTKV<br>DKKIEPRVPITQNPCPPLKECPPCAAPDLLGGPSVFIFPPKIKDVLMISLSPMVTCVVVDVSEDDPDVQISWFVNNV<br>EVHTAQTQTHREDYNSTLRVVSALPIQHQDWMSGKEFKCKVNNRALPSPIEKTISKPRGPVRAPQVYVLPPPAEE<br>MTKKEFSLTCMITGFLPAEIAVDWTSNGRTEQNYKNTATVLDSDGSYFMYSKLRVQKSTWERGSLFACSVVHEGL<br>HNHLTTKTISRSLGK

```
IgLC1      GCCAGCCCAAGTCTTCGCCATCAGTCACCCTGTTTCCACCTTCCTCTGAAGAGCTCGAGA     60
IgLC2      GTCAGCCCAAGTCCACTCCCACTCTCACCGTGTTTCCACCTTCCTCTGAGGAGCTCAAGG     60
IgLC3      GTCAGCCCAAGTCCACTCCCACACTCACCATGTTTCCACCTTCCCCTGAGGAGCTCCAGG     60
IgLC4      GCCAACCCAAGGCTACACCCTCAGTTAATCTGTTCCCACCTTCCTCTGAAGAGCTCAAGA     60
           * ** ****** *  * **  *  * *   **** ********* **** ****** **

IgLC1      CTAACAAGGCCACACTGGTGTGTACGATCACTGATTTCTACCCAGGTGTGGTGACAGTGG     120
IgLC2      AAAACAAAGCCACACTGGTGTGTCTGATTTCCAACTTTTCCCCGAGTGGTGTGACAGTGG     120
IgLC3      AAAACAAAGCCACACTCGTGTGTCTGATTTCCAATTTTTCCCCAAGTGGTGTGACAGTGG     120
IgLC4      CTAAAAAGGCCACACTGGTGTGTATGATCACTGAGTTCTACGCAGCTGCTGTGAGAGTGG     120
             ** ** ******** ******  ***  *  * ** * * *   **  **** *****

IgLC1      ACTGGAAGGTAGATGGTACCCCTGTCACTCAGGGTATGGAGACAACCCAGCCTTCCAAAC     180
IgLC2      CCTGGAAGGCAAATGGTACACCTATCACCCAGGGTGTGGACACTTCAAATCCCACCAAAG     180
IgLC3      CCTGGAAGGCAAATGGTACACCTATCACCCAGGGTGTGGACACTTCAAATCCCACCAAAG     180
IgLC4      CCTGGAAGGCAGATGGTACCCCTTTCACTCAGGGTGTAGAGACTACCCAGCCTCCCAAAC     180
            ******** * ******* *** **** ****** * ** **  *  * **  *****

IgLC1      AGAGCAACAACAAGTACATGGCTAGCAGCTACCTGACCCTGACAGCAAGAGCATGGGAAA     240
IgLC2      AGG---GCAACAAGTTCATGGCCAGCAGCTTCCTACATTTGACATCGGACCAGTGGAGAT     237
IgLC3      AGG---ACAACAAGTACATGGCCAGCAGCTTCTTACATTTGACATCGGACCAGTGGAGAT     237
IgLC4      AGAGGGACAA------CATGGCTAGCAGTTACCTGCTCTTCACAGCAGAAGCGTGGGAAT     234
           **     ***      ****** ***** * * *     * *** *       ***  *

IgLC1      GGCATAGCAGTTACAGCTGCCAGGTCACTCATGAAGGTCACACTGTGGAGAAGAGTTTGT     300
IgLC2      CTCACAACAGTTTTACCTGTCAAGTTACACATGAAGGGGACACTGTGGAGAAGAGTCTGT     297
IgLC3      CTCACAACAGTTTTACCTGCCAAGTTACACATGAAGGGGACACTGTGGAGAAGAGTCTGT     297
IgLC4      CTCATAGCAGTTACAGCTGCCATGTCACTCATGAAGGGCAACATGTGGAGAAGAGTTTGT     294
             ** * *****  * *** ** ** ** ********  *   ************* ***

IgLC1      CCCGTGCTGACTGTTCCTAG  320
IgLC2      CTCCTGCAGAATGTCTCTAA  317
IgLC3      CTCCTGCAGAATGTCTC---  314
IgLC4      CCCGTGCTGAGTGTTCC---  311
           * * *** ** ***  *
```

Figure S1. DNA alignment from constant region of all mouse IgG lambda light chains. Bases are marked with an asterisk if they are conserved in all subclasses. Binding sites of primers for reverse transcription and PCR amplification are marked in yellow and blue. The underlined sequence represents primer complement site. The sequence motifs with greatest differences suitable for lambda light chain determination are highlighted in green.

```
Length=602
                                                                                                      Score     E
Sequences producing significant alignments:                                                          (Bits)  Value

IGHV2-6*01germline gene                                                                               441     1e-125
IGHV2-6-8*01germline gene                                                                             441     1e-125
IGHV2-9*01germline gene                                                                               417     1e-118
IGHD1-1*01germline gene                                                                               12.2    41
IGHD1-1*02germline gene                                                                               12.2    41
IGHJ3*01germline gene                                                                                 81.4    1e-19
IGHJ3*02germline gene                                                                                 69.9    4e-16
IGHJ2*01germline gene                                                                                 21.8    0.12


Domain classification requested: imgt
```

V-(D)-J rearrangement summary for query sequence (multiple equivalent top matches, if present, are separated by a comma):

Top V gene match	Top D gene match	Top J gene match	Chain type	stop codon	V-J frame	Productive	Strand	V frame shift
IGHV2-6*01,IGHV2-6-8*01	IGHD1-1*01,IGHD1-1*02	IGHJ3*01	VH	No	In-frame	Yes	+	No

V-(D)-J junction details based on top germline gene matches:

V region end	V-D junction*	D region	D-J junction*	J region start
GTCAG	A	TACGGT	GGTGGAG	GGTTT

*: Overlapping nucleotides may exist at V-D-J junction (i.e, nucleotides that could be assigned to either rearranging gene). Such nucleotides are indicated inside a parenthesis (i.e., (TACAT)) but are not included under the V, D or J gene itself.

Sub-region sequence details:

	Nucleotide sequence	Translation	Start	End
CDR3	GTCAGATACGGTGGTGGAGGGTTTGCTTAC	VRYGGGGFAY	404	433

Sub-region sequence details:

	Nucleotide sequence	Translation	Start	End
CDR3	GTCAGATACGGTGGTGGAGGGTTTGCTTAC	VRYGGGGFAY	404	433

Alignment summary between query and top germline V gene hit:

	from	to	length	matches	mismatches	gaps	identity(%)
FR1-IMGT	119	193	75	75	0	0	100
CDR1-IMGT	194	217	24	22	2	0	91.7
FR2-IMGT	218	268	51	50	1	0	98
CDR2-IMGT	269	289	21	21	0	0	100
FR3-IMGT	290	403	114	114	0	0	100
CDR3-IMGT (germline)	404	408	5	4	1	0	80
Total			290	286	4	0	98.6

Alignments

```
                                              <--------------------------------FR1-IMGT---------------------------------><-------CDR1-IM
                                               Q  V  Q  L  K  E  S  G  P  G  L  V  A  P  S  Q  S  L  S  I  T  C  T  V  S  G  F  S  L  S
                           Query_1       119  CAGGTGCAGCTGAAGGAGTCAGGACCTGGCCTGGTGGCGCCCTCACAGAGCCTGTCCATCACATGCACTGTCTCTGGGTTCTCATTAAGC  208
V  98.6% (286/290)  IGHV2-6*01      1    ........................................................................................C.  90
                                               Q  V  Q  L  K  E  S  G  P  G  L  V  A  P  S  Q  S  L  S  I  T  C  T  V  S  G  F  S  L  T
V  98.6% (286/290)  IGHV2-6-8*01    1    ........................................................................................C.  90
V  96.2% (278/289)  IGHV2-9*01      1    ..............................................................T.................T.......C.  90

                                              GT------><--------------------FR2-IMGT---------------------><-----CDR2-IMGT-----><--------
                                               S  Y  V  V  D  W  V  R  Q  S  P  G  K  G  L  E  W  L  G  V  I  W  G  V  G  S  T  N  Y  N
                           Query_1       209  AGCTATGTTGTAGACTGGGTTCGCCAGTCTCCAGGAAAGGGTCTGGAATGGCTGGGAGTAATATGGGGTGTTGGAAGCACAAATTATAAT  298
V  98.6% (286/290)  IGHV2-6*01      91   ......G........................................G..........................................  180
                                               S  Y  G  V  D  W  V  R  Q  S  P  G  K  G  L  E  W  L  G  V  I  W  G  V  G  S  T  N  Y  N
V  98.6% (286/290)  IGHV2-6-8*01    91   ......G........................................G..........................................  180
V  96.2% (278/289)  IGHV2-9*01      91   ......G....................C...................G......................G...................  180

                                              -----------------------------------------FR3-IMGT-----------------------------------------
                                               S  A  L  K  S  R  L  S  I  S  K  D  N  S  K  S  Q  V  F  L  K  M  N  S  L  Q  T  D  D  T
                           Query_1       299  TCAGCTCTCAAATCCAGACTGAGCATCAGCAAGGACAACTCCAAGAGCCAAGTTTTCTTAAAAATGAACAGTCTGCAAACTGATGACACA  388
V  98.6% (286/290)  IGHV2-6*01      181  ..........................................................................................  270
                                               S  A  L  K  S  R  L  S  I  S  K  D  N  S  K  S  Q  V  F  L  K  M  N  S  L  Q  T  D  D  T
V  98.6% (286/290)  IGHV2-6-8*01    181  ..........................................................................................  270
V  96.2% (278/289)  IGHV2-9*01      181  ..........TG....................A.........................................................  270

                                              --------------><----------CDR3-IMGT---------><-----------FR4-IMGT------------>
                                               A  M  Y  Y  C  V  R  Y  G  G  G  G  F  A  Y  W  G  Q  G  T  L  V  T  V  S  A
                           Query_1       389  GCCATGTACTACTGTGTCAGATACGGTGGTGGAGGGTTTGCTTACTGGGGCCAGGGGACTCTGGTCACTGTCTCTGCAG  467
V  98.6% (286/290)  IGHV2-6*01      271  ................C...-----------------------------------------------------------  290
                                               A  M  Y  Y  C  A
V  98.6% (286/290)  IGHV2-6-8*01    271  ................C...-----------------------------------------------------------  290
V  96.2% (278/289)  IGHV2-9*01      271  ................C..------------------------------------------------------------  289
D  100.0% (6/6)     IGHD1-1*01      9    ---------------------......----------------------------------------------------  14
D  100.0% (6/6)     IGHD1-1*02      11   --------------------------......-----------------------------------------------  16
D  100.0% (6/6)     IGHD1-1*02      12   ------------------------......-------------------------------------------------  17
J  97.8% (44/45)    IGHJ3*01        4    ----------------------------------...................A.........................  48
J  93.3% (42/45)    IGHJ3*02        4    ----------------------------------......G...G........A.........................  48
J  100.0% (11/11)   IGHJ2*01        12   ------------------------------------------...........--------------------------  22
```

Figure S2: Data output for IgBLAST-generated analysis of mouse anti-CCMV monoclonal antibody (BAM-CCMV-29-81) heavy chain variable sequence. The summary of V-, D- and J- genes rearrangement, discovered identity [%] for the closest-matching genes as well as the DNA and amino acid matches are shown.

Length=559

Sequences producing significant alignments:	Score (Bits)	E Value
IGKV3-12*01germline gene	444	1e-126
IGKV3-7*02germline gene	413	3e-117
IGKV3-7*01germline gene	410	3e-116
IGKJ2*01germline gene	69.9	4e-16
IGKJ2*03germline gene	54.5	2e-11
IGKJ2*02germline gene	48.8	9e-10

Domain classification requested: imgt

V-(D)-J rearrangement summary for query sequence (multiple equivalent top matches, if present, are separated by a comma):

Top V gene match	Top J gene match	Chain type	stop codon	V-J frame	Productive	Strand	V frame shift
IGKV3-12*01	IGKJ2*01	VK	Yes	Out-of-frame	No	+	No

V-(D)-J junction details based on top germline gene matches:

V region end	V-J junction*	J region start
AGCTT		ACACG

*: Overlapping nucleotides may exist at V-D-J junction (i.e, nucleotides that could be assigned to either rearranging gene). Such nucleotides are indicated inside a parenthesis (i.e., (TACAT)) but are not included under the V, D or J gene itself.

Sub-region sequence details:

	Nucleotide sequence	Translation	Start	End
CDR3	CAGCACATTAGGGAGCTTACACG	QHIRELTR	391	413

Alignment summary between query and top germline V gene hit:

	from	to	length	matches	mismatches	gaps	identity(%)
FR1-IMGT	115	192	78	77	1	0	98.7
CDR1-IMGT	193	222	30	30	0	0	100
FR2-IMGT	223	273	51	49	2	0	96.1
CDR2-IMGT	274	282	9	8	1	0	88.9
FR3-IMGT	283	390	108	108	0	0	100
CDR3-IMGT (germline)	391	408	18	17	1	0	94.4
Total			294	289	5	0	98.3

Alignments

```
                                           <----------------------------------FR1-IMGT----------------------------------><----------C
                                            D  I  V  L  T  Q  S  P  A  S  L  A  V  S  L  G  Q  R  A  T  I  S  Y  R  A  S  K  S  V  S
                         Query_1      115  GACATTGTGCTGACACAGTCTCCTGCTTCCTTAGCTGTATCTCTGGGGCAGAGGGCCACCATCTCATACAGGGCCAGCAAAAGTGTCAGT  204
V  98.3% (289/294)       IGKV3-12*01  1    ...................................................................G......................  90
                                            D  I  V  L  T  O  S  P  A  S  L  A  V  S  L  G  O  R  A  T  I  S  C  R  A  S  K  S  V  S
V  94.9% (279/294)       IGKV3-7*02   1    ...................................................................G...........C..........  90
V  94.6% (278/294)       IGKV3-7*01   1    ...........A.......................................................G...........C..........  90

                                           DR1-IMGT---------><--------------------FR2-IMGT---------------------><CDR2-IM><-----------
                                            T  S  G  Y  S  Y  M  H  W  N  Q  Q  K  P  G  Q  P  P  R  L  L  I  Y  L  V  S  N  L  E  S
                         Query_1      205  ACATCTGGCTATAGTTATATGCACTGGAACCAACAGAAACCAGGACAGCCACCCAGACTCCTCATCTATCTTGTATCCAACCTAGAATCT  294
V  98.3% (289/294)       IGKV3-12*01  91   ...........................T...........................A.................C................  180
                                            T  S  G  Y  S  Y  M  H  W  Y  Q  Q  K  P  G  Q  P  P  K  L  L  I  Y  L  A  S  N  L  E  S
V  94.9% (279/294)       IGKV3-7*02   91   ......A....................T...........................A..........A.GTA..C................  180
V  94.6% (278/294)       IGKV3-7*01   91   ......A....................T...........................A..........A.GTA..C................  180

                                           -------------------------------------FR3-IMGT---------------------------------------------
                                            G  V  P  A  R  F  S  G  S  G  S  G  T  D  F  T  L  N  I  H  P  V  E  E  E  D  A  A  T  Y
                         Query_1      295  GGGGTCCCTGCCAGGTTCAGTGGCAGTGGGTCTGGGACAGACTTCACCCTCAACATCCATCCTGTGGAGGAGGAGGATGCTGCAACCTAT  384
V  98.3% (289/294)       IGKV3-12*01  181  ..........................................................................................  270
                                            G  V  P  A  R  F  S  G  S  G  S  G  T  D  F  T  L  N  I  H  P  V  E  E  E  D  A  A  T  Y
V  94.9% (279/294)       IGKV3-7*02   181  ..............................................................................A.......A...  270
V  94.6% (278/294)       IGKV3-7*01   181  ..............................................................................A.......A...  270

                                           -----><------CDR3-IMGT------><----------FR4-IMGT---------->
                                            Y  C  Q  H  I  R  E  L  T  R  S  E  G  G  P  S  W  K  *  N
                         Query_1      385  TACTGTCAGCACATTAGGGAGCTTACACGTTCGGAGGGGGGACCAAGCTGGAAATAAAAC  444
V  98.3% (289/294)       IGKV3-12*01  271  .............G..........------------------------------------  294
                                            Y  C  Q  H  S  R  E  L
V  94.9% (279/294)       IGKV3-7*02   271  .............G.T.....A..------------------------------------  294
V  94.6% (278/294)       IGKV3-7*01   271  .............G.T.....A..------------------------------------  294
J  100.0% (36/36)        IGKJ2*01     4    ------------------------....................................  39
J  94.1% (32/34)         IGKJ2*03     6    --------------------------.........TC.......................  39
J  91.2% (31/34)         IGKJ2*02     6    --------------------------.........TC..................G....  39
```

Figure S3: Data output for IgBLAST-generated analysis of mouse anti-CCMV monoclonal antibody (BAM-CCMV-29-81) unfunctional κ-chain variable sequence. The summary of V-, D- and J- genes rearrangement, discovered identity [%] for the closest-matching genes as well as the DNA and amino acid matches are shown. Frame region 4 contains an early stop codon (*) due to a frameshift mutation.

Length=581

Sequences producing significant alignments:

	Score (Bits)	E Value
IGKV4-61*01germline gene	436	3e-124
IGKV4-52*01germline gene	408	9e-116
IGKV4-54*01germline gene	408	9e-116
IGKJ1*01germline gene	69.9	4e-16
IGKJ1*02germline gene	64.1	2e-14
IGKJ2*01germline gene	43.0	5e-08

Domain classification requested: imgt

V-(D)-J rearrangement summary for query sequence (multiple equivalent top matches, if present, are separated by a comma):

Top V gene match	Top J gene match	Chain type	stop codon	V-J frame	Productive	Strand	V frame shift
IGKV4-61*01	IGKJ1*01	VK	No	In-frame	Yes	+	No

V-(D)-J junction details based on top germline gene matches:

V region end	V-J junction*	J region start
CCCAC		GGACG

*: Overlapping nucleotides may exist at V-D-J junction (i.e, nucleotides that could be assigned to either rearranging gene). Such nucleotides are indicated inside a parenthesis (i.e., (TACAT)) but are not included under the V, D or J gene itself.

Sub-region sequence details:

	Nucleotide sequence	Translation	Start	End
CDR3	CAGCAGTATCATAGTTACCCACGGACG	QQYHSYPRT	409	435

Alignment summary between query and top germline V gene hit:

	from	to	length	matches	mismatches	gaps	identity(%)
FR1-IMGT	148	225	78	78	0	0	100
CDR1-IMGT	226	240	15	14	1	0	93.3
FR2-IMGT	241	291	51	50	1	0	98
CDR2-IMGT	292	300	9	9	0	0	100
FR3-IMGT	301	408	108	108	0	0	100
CDR3-IMGT (germline)	409	430	22	22	0	0	100
Total			283	281	2	0	99.3

Alignments

```
                                      <----------------------------------FR1-IMGT----------------------------------><--CDR1-IMGT
                                       Q  I  V  L  T  Q  S  P  A  I  M  S  A  S  P  G  E  K  V  T  I  S  C  S  A  S  S  S  I  S 
                    Query_1      148  CAAATTGTTCTCACCCAGTCTCCAGCAATCATGTCTGCATCTCCAGGGGAGAAGGTCACCATATCCTGCAGTGCCAGCTCAAGTATAAGT  237
V  99.3% (281/283)  IGKV4-61*01  1    ....................................................................................G.....  90
                                       Q  I  V  L  T  Q  S  P  A  I  M  S  A  S  P  G  E  K  V  T  I  S  C  S  A  S  S  S  V  S 
V  96.1% (272/283)  IGKV4-52*01  1    ..............................C...............................GA....................G.....  90
V  96.1% (272/283)  IGKV4-54*01  1    ..............................C...............................GA....................G.....  90

                                      --><--------------------FR2-IMGT---------------------><CDR2-IM><--------------------------
                                       Y  M  Y  W  Y  Q  Q  K  P  G  S  S  P  K  S  W  I  Y  R  T  S  N  L  A  S  G  V  P  A  R 
                    Query_1      238  TACATGTACTGGTACCAGCAGAAGCCAGGATCCTCCCCCAAATCCTGGATTTATCGCACATCCAACCTGGCTTCTGGAGTCCCTGCTCGC  327
V  99.3% (281/283)  IGKV4-61*01  91   ..........................................C...............................................  180
                                       Y  M  Y  W  Y  Q  Q  K  P  G  S  S  P  K  P  W  I  Y  R  T  S  N  L  A  S  G  V  P  A  R 
V  96.1% (272/283)  IGKV4-52*01  91   .........A.........................A......C...........G...................................  180
V  96.1% (272/283)  IGKV4-54*01  91   .........A.........................A......C...........G...................................  180

                                      -----------------------FR3-IMGT-------------------------------------------------><--------
                                       F  S  G  S  G  S  G  T  S  Y  S  L  T  I  S  S  M  E  A  E  D  A  A  T  Y  Y  C  Q  Q  Y 
                    Query_1      328  TTCAGTGGCAGTGGGTCTGGGACCTCTTACTCTCTCACAATCAGCAGCATGGAGGCTGAAGATGCTGCCACTTATTACTGCCAGCAGTAT  417
V  99.3% (281/283)  IGKV4-61*01  181  ..........................................................................................  270
                                       F  S  G  S  G  S  G  T  S  Y  S  L  T  I  S  S  M  E  A  E  D  A  A  T  Y  Y  C  Q  Q  Y 
V  96.1% (272/283)  IGKV4-52*01  181  ..............A..............T........................................................A...  270
V  96.1% (272/283)  IGKV4-54*01  181  ..............A..............T........................................................A...  270

                                      CDR3-IMGT--------><----------FR4-IMGT---------->
                                       H  S  Y  P  R  T  F  G  G  G  T  K  L  E  I  K 
                    Query_1      418  CATAGTTACCCACGGACGTTCGGTGGAGGCACCAAGCTGGAAATCAAAC  466
V  99.3% (281/283)  IGKV4-61*01  271  .............------------------------------------  283
                                       H  S  Y  P 
V  96.1% (272/283)  IGKV4-52*01  271  .............------------------------------------  283
V  96.1% (272/283)  IGKV4-54*01  271  .............------------------------------------  283
J  100.0% (36/36)   IGKJ1*01     3    -------------....................................  38
J  100.0% (33/33)   IGKJ1*02     3    --------------.................................--  35
J  88.2% (30/34)    IGKJ2*01     6    ---------------........A..G..G..............A....  39
```

Figure S4: Data output for IgBLAST-generated analysis of mouse anti-CCMV monoclonal antibody (BAM-CCMV-29-81) functional κ- chain variable sequence. The summary of V-, D- and J- genes rearrangement, discovered identity [%] for the closest-matching genes as well as the DNA and amino acid matches are shown.

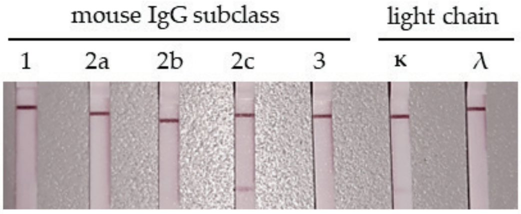

Figure S5: Determination of IgG subclass from mouse monoclonal antibody of clone BAM-CCMV-29-81 in the undiluted hybridoma supernatant.

```
sample      GAAATCAAACGGGCTGATGCTGCACCAACTGTATCCATCTTCCCACCATCCAGTGAGCAGTTAACATCTGGAGGTGCCTCAGTCGTGTGCTTCTTGAACA 100
mIgK        ----------GGGCTGATGCTGCACCAACTGTATCCATCTTCCCACCATCCAGTGAGCAGTTAACATCTGGAGGTGCCTCAGTCGTGTGCTTCTTGAACA 100
            ****************************************************************************************************

sample      ACTTCTACCCCAAAGACATCAATGTCAAGTGGAAGATTGATGGCAGTGAACGACAAAATGGCGTCCTGAACAGTTGGACTGATCAGGACAGCAAAGACAG 200
mIgK        ACTTCTACCCCAAAGACATCAATGTCAAGTGGAAGATTGATGGCAGTGAACGACAAAATGGCGTCCTGAACAGTTGGACTGATCAGGACAGCAAAGACAG 200
            ****************************************************************************************************

sample      CACCTACAGCATGAGCAGCACCCTCACGTTGACCAAGGACGAGTATGAACGACATAACAGCTATACCTGTGAGGCCACTCACAAGACATCAACTTCACCC 300
mIgK        CACCTACAGCATGAGCAGCACCCTCACGTTGACCAAGGACGAGTATGAACGACATAACAGCTATACCTGTGAGGCCACTCACAAGACATCAACTTCACCC 300
            ****************************************************************************************************

sample      ATTGTCAAGAGCTTCAACAGGAATGAGTGTTAGAGACAAAGGTCCTGAGACGCCACCACCAGCTCCCCAGCTCCATCCTATCTTCCCTTCTAAGGTCTTG 400
mIgK        ATTGTCAAGAGCTTCAACAGGAATGAGTGT---------------------------------------------------------------------- 400
            ****************************************************************************************************
```

Figure S6. DNA alignment from constant regions of mouse κ-chain with sequencing results of mouse monoclonal antibody from clone BAM-CCMV-29-81 light chain. Matching bases are marked with an asterisk.

```
sample           CCAAAACAACAGCCCCATCGGTCTATCCACTGGCCCCTGTGTGTGGAGGTACAACTGGCTCCTCGGTGACTCTAGGATGCCTGGTCAAGGGTTATTTCCC 100
mIg2C-CH1        CCAAAACAACAGCCCCATCGGTCTATCCACTGGCCCCTGTGTGTGGAGGTACAACTGGCTCCTCGGTGACTCTAGGATGCCTGGTCAAGGGTTATTTCCC 100
mIg2C-Hinge      ---------------------------------------------------------------------------------------------------- 100
mIg2C-CH2        ---------------------------------------------------------------------------------------------------- 100
mIg2C-CH3-CHS    ---------------------------------------------------------------------------------------------------- 100
                 ****************************************************************************************************

sample           TGAGCCAGTGACCTTGACCTGGAACTCTGGATCCCTGTCCAGTGGTGTGCACACCTTCCCAGCTCTCCTGCAGTCTGGCCTCTACACCCTCAGCAGCTCA 200
mIg2C-CH1        TGAGCCAGTGACCTTGACCTGGAACTCTGGATCCCTGTCCAGTGGTGTGCACACCTTCCCAGCTCTCCTGCAGTCTGGCCTCTACACCCTCAGCAGCTCA 200
mIg2C-Hinge      ---------------------------------------------------------------------------------------------------- 200
mIg2C-CH2        ---------------------------------------------------------------------------------------------------- 200
mIg2C-CH3-CHS    ---------------------------------------------------------------------------------------------------- 200
                 ****************************************************************************************************

sample           GTGACTGTAACCTCGAACACCTGGCCCAGCCAGACCATCACCTGCAATGTGGCCCACCCGGCAAGCAGCACCAAAGTGGACAAGAAAATTGAGCCCAGAG 300
mIg2C-CH1        GTGACTGTAACCTCGAACACCTGGCCCAGCCAGACCATCACCTGCAATGTGGCCCACCCGGCAAGCAGCACCAAAGTGGACAAGAAAATTG--------- 300
mIg2C-Hinge      -------------------------------------------------------------------------------------------AGCCCAGAG 300
mIg2C-CH2        ---------------------------------------------------------------------------------------------------- 300
mIg2C-CH3-CHS    ---------------------------------------------------------------------------------------------------- 300
                 ****************************************************************************************************

sample           TGCCCATAACACAGAACCCCTGTCCTCCACTCAAAGAGTGTCCCCCATGCGCAGCTCCAGACCTCTTGGGTGGACCATCCGTCTTCATCTTCCCTCCAAA 400
mIg2C-CH1        ---------------------------------------------------------------------------------------------------- 400
mIg2C-Hinge      TGCCCATAACACAGAACCCCTGTCCTCCACTCAAAGAGTGTCCCCCATGCGCAG---------------------------------------------- 400
mIg2C-CH2        ------------------------------------------------------CTCCAGACCTCTTGGGTGGACCATCCGTCTTCATCTTCCCTCCAAA 400
mIg2C-CH3-CHS    ---------------------------------------------------------------------------------------------------- 400
                 ****************************************************************************************************

sample           GATCAAGGATGTACTCATGATCTCCCTGAGCCCCATGGTCACATGTGTGGTGGTGGATGTGAGCGAGGATGACCCAGACGTCCAGATCAGCTGGTTTGTG 500
mIg2C-CH1        ---------------------------------------------------------------------------------------------------- 500
mIg2C-Hinge      ---------------------------------------------------------------------------------------------------- 500
mIg2C-CH2        GATCAAGGATGTACTCATGATCTCCCTGAGCCCCATGGTCACATGTGTGGTGGTGGATGTGAGCGAGGATGACCCAGACGTCCAGATCAGCTGGTTTGTG 500
mIg2C-CH3-CHS    ---------------------------------------------------------------------------------------------------- 500
                 ****************************************************************************************************

sample           AACAACGTGGAAGTACACACAGCTCAGACACAAACCCATAGAGAGGATTACAACAGTACTCTCCGGGTGGTCAGTGCCCTCCCCATCCAGCACCAGGACT 600
mIg2C-CH1        ---------------------------------------------------------------------------------------------------- 600
mIg2C-Hinge      ---------------------------------------------------------------------------------------------------- 600
mIg2C-CH2        AACAACGTGGAAGTACACACAGCTCAGACACAAACCCATAGAGAGGATTACAACAGTACTCTCCGGGTGGTCAGTGCCCTCCCCATCCAGCACCAGGACT 600
mIg2C-CH3-CHS    ---------------------------------------------------------------------------------------------------- 600
                 ****************************************************************************************************

sample           GGATGAGTGGCAAGGAGTTCAAATGCAAGGTCAACAACAGAGCCCTCCCATCCCCCATCGAGAAAACCATCTCAAAACCCAGAGGGCCAGTAAGAGCTCC 700
mIg2C-CH1        ---------------------------------------------------------------------------------------------------- 700
mIg2C-Hinge      ---------------------------------------------------------------------------------------------------- 700
mIg2C-CH2        GGATGAGTGGCAAGGAGTTCAAATGCAAGGTCAACAACAGAGCCCTCCCATCCCCCATCGAGAAAACCATCTCAAAACCCAGAG---------------- 700
mIg2C-CH3-CHS    ------------------------------------------------------------------------------------GGCCAGTAAGAGCTCC 700
                 ****************************************************************************************************

sample           ACAGGTATATGTCTTGCCTCCACCAGCAGAAGAGATGACTAAGAAAGAGTTCAGTCTGACCTGCATGATCACAGGCTTCTTACCTGCCGAAATTGCTGTG 800
mIg2C-CH1        ---------------------------------------------------------------------------------------------------- 800
mIg2C-Hinge      ---------------------------------------------------------------------------------------------------- 800
mIg2C-CH2        ---------------------------------------------------------------------------------------------------- 800
mIg2C-CH3-CHS    ACAGGTATATGTCTTGCCTCCACCAGCAGAAGAGATGACTAAGAAAGAGTTCAGTCTGACCTGCATGATCACAGGCTTCTTACCTGCCGAAATTGCTGTG 800
                 ****************************************************************************************************

sample           GACTGGACCAGCAATGGGCGTACAGAGCAAAACTACAAGAACACCGCAACAGTCCTGGACTCTGATGGTTCTTACTTCATGTACAGCAAGCTCAGAGTAC 900
mIg2C-CH1        ---------------------------------------------------------------------------------------------------- 900
mIg2C-Hinge      ---------------------------------------------------------------------------------------------------- 900
mIg2C-CH2        ---------------------------------------------------------------------------------------------------- 900
mIg2C-CH3-CHS    GACTGGACCAGCAATGGGCGTACAGAGCAAAACTACAAGAACACCGCAACAGTCCTGGACTCTGATGGTTCTTACTTCATGTACAGCAAGCTCAGAGTAC 900
                 ****************************************************************************************************

sample           AAAAGAGCACTTGGGAAAGAGGAAGTCTTTTCGCCTGCTCAGTGGTCCACGAGGGTCTGCACAATCACCTTACGACTAAGACCATCTCCCGGTCTCTGGG 1000
mIg2C-CH1        ---------------------------------------------------------------------------------------------------- 1000
mIg2C-Hinge      ---------------------------------------------------------------------------------------------------- 1000
mIg2C-CH2        ---------------------------------------------------------------------------------------------------- 1000
mIg2C-CH3-CHS    AAAAGAGCACTTGGGAAAGAGGAAGTCTTTTCGCCTGCTCAGTGGTCCACGAGGGTCTGCACAATCACCTTACGACTAAGACCATCTCCCGGTCTCTGGG 1000
                 ****************************************************************************************************

sample           TAAA---------------------------------------------------------------------------------                1004
mIg2C-CH1        -------------------------------------------------------------------------------------                1004
mIg2C-Hinge      -------------------------------------------------------------------------------------                1004
mIg2C-CH2        -------------------------------------------------------------------------------------                1004
mIg2C-CH3-CHS    TAAA---------------------------------------------------------------------------------                1004
                 ****
```

Figure S7. DNA alignment from constant regions C_H1, Hinge, C_H2 and C_H3 of mouse heavy chain IgG2c subclass with sequencing results of mouse anti-CCMV monoclonal antibody (BAM-CCMV-29-81) constant domains. Matching bases are marked with an asterisk.

(A)

```
RNA_SEQ      CAGGTGCAGCTGAAGGAGTCAGGACCTGGCCTGGTGGCGCCCTCACAGAGCCTGTCCATCACATGCACTGTCTCTGGGTTCTCATTAAGCAGCTATGTTG 100
BAM          CAGGTGCAGCTGAAGGAGTCAGGACCTGGCCTGGTGGCGCCCTCACAGAGCCTGTCCATCACATGCACTGTCTCTGGGTTCTCATTAAGCAGCTATGTTG 100
             ****************************************************************************************************

RNA_SEQ      TAGACTGGGTTCGCCAGTCTCCAGGAAAGGGTCTGGAATGGCTGGGAGTAATATGGGGTGTTGGAAGCACAAATTATAATTCAGCTCTCAAATCCAGACT 200
BAM          TAGACTGGGTTCGCCAGTCTCCAGGAAAGGGTCTGGAATGGCTGGGAGTAATATGGGGTGTTGGAAGCACAAATTATAATTCAGCTCTCAAATCCAGACT 200
             ****************************************************************************************************

RNA_SEQ      GAGCATCAGCAAGGACAACTCCAAGAGCCAAGTTTTCTTAAAAATGAACAGTCTGCAAACTGATGACACAGCCATGTACTACTGTGTCAGATACGGTGGT 300
BAM          GAGCATCAGCAAGGACAACTCCAAGAGCCAAGTTTTCTTAAAAATGAACAGTCTGCAAACTGATGACACAGCCATGTACTACTGTGTCAGATACGGTGGT 300
             ****************************************************************************************************

RNA_SEQ      GGAGGGTTTGCTTACTGGGGCCAGGGGACTCTGGTCACTGTCTCTGCAGCCAAAACAACAGCCCCATCGGTCTATCCACTGGCCCCTGTGTGTGGAGGTA 400
BAM          GGAGGGTTTGCTTACTGGGGCCAGGGGACTCTGGTCACTGTCTCTGCAGCCAAAACAACAGCCCCATCGGTCTATCCACTGGCCCCTGTGTGTGGAGGTA 400
             ****************************************************************************************************

RNA_SEQ      CAACTGGCTCCTCGGTGACTCTAGGATGCCTGGTCAAGGGTTATTTCCCTGAGCCAGTGACCTTGACCTGGAACTCTGGATCCCTGTCCAGTGGTGTGCA 500
BAM          CAACTGGCTCCTCGGTGACTCTAGGATGCCTGGTCAAGGGTTATTTCCCTGAGCCAGTGACCTTGACCTGGAACTCTGGATCCCTGTCCAGTGGTGTGCA 500
             ****************************************************************************************************

RNA_SEQ      CACCTTCCCAGCTCTCCTGCAGTCTGGCCTCTACACCCTCAGCAGCTCAGTGACTGTAACCTCGAACACCTGGCCCAGCCAGACCATCACCTGCAATGTG 600
BAM          CACCTTCCCAGCTCTCCTGCAGTCTGGCCTCTACACCCTCAGCAGCTCAGTGACTGTAACCTCGAACACCTGGCCCAGCCAGACCATCACCTGCAATGTG 600
             ****************************************************************************************************

RNA_SEQ      GCCCACCCGGCAAGCAGCACCAAAGTGGACAAGAAAATTGAGCCCAGAGTGCCCATAACACAGAACCCCTGTCCTCCACTCAAAGAGTGTCCCCCATGCG 700
BAM          GCCCACCCGGCAAGCAGCACCAAAGTGGACAAGAAAATTGAGCCCAGAGTGCCCATAACACAGAACCCCTGTCCTCCACTCAAAGAGTGTCCCCCATGCG 700
             ****************************************************************************************************

RNA_SEQ      CAGCTCCAGACCTCTTGGGTGGACCATCCGTCTTCATCTTCCCTCCAAAGATCAAGGATGTACTCATGATCTCCCTGAGCCCCATGGTCACATGTGTGGT 800
BAM          CAGCTCCAGACCTCTTGGGTGGACCATCCGTCTTCATCTTCCCTCCAAAGATCAAGGATGTACTCATGATCTCCCTGAGCCCCATGGTCACATGTGTGGT 800
             ****************************************************************************************************

RNA_SEQ      GGTGGATGTGAGCGAGGATGACCCAGACGTCCAGATCAGCTGGTTTGTGAACAACGTGGAAGTACACACAGCTCAGACACAAACCCATAGAGAGGATTAC 900
BAM          GGTGGATGTGAGCGAGGATGACCCAGACGTCCAGATCAGCTGGTTTGTGAACAACGTGGAAGTACACACAGCTCAGACACAAACCCATAGAGAGGATTAC 900
             ****************************************************************************************************

RNA_SEQ      AACAGTACTCTCCGGGTGGTCAGTGCCCTCCCCATCCAGCACCAGGACTGGATGAGTGGCAAGGAGTTCAAATGCAAGGTCAACAACAGAGCCCTCCCAT 1000
BAM          AACAGTACTCTCCGGGTGGTCAGTGCCCTCCCCATCCAGCACCAGGACTGGATGAGTGGCAAGGAGTTCAAATGCAAGGTCAACAACAGAGCCCTCCCAT 1000
             ****************************************************************************************************

RNA_SEQ      CCCCCATCGAGAAAACCATCTCAAAACCCAGAGGGCCAGTAAGAGCTCCACAGGTATATGTCTTGCCTCCACCAGCAGAAGAGATGACTAAGAAAGAGTT 1100
BAM          CCCCCATCGAGAAAACCATCTCAAAACCCAGAGGGCCAGTAAGAGCTCCACAGGTATATGTCTTGCCTCCACCAGCAGAAGAGATGACTAAGAAAGAGTT 1100
             ****************************************************************************************************

RNA_SEQ      CAGTCTGACCTGCATGATCACAGGCTTCTTACCTGCCGAAATTGCTGTGGACTGGACCAGCAATGGGCGTACAGAGCAAAACTACAAGAACACCGCAACA 1200
BAM          CAGTCTGACCTGCATGATCACAGGCTTCTTACCTGCCGAAATTGCTGTGGACTGGACCAGCAATGGGCGTACAGAGCAAAACTACAAGAACACCGCAACA 1200
             ****************************************************************************************************

RNA_SEQ      GTCCTGGACTCTGATGGTTCTTACTTCATGTACAGCAAGCTCAGAGTACAAAAGAGCACTTGGGAAAGAGGAAGTCTTTTCGCCTGCTCAGTGGTCCACG 1300
BAM          GTCCTGGACTCTGATGGTTCTTACTTCATGTACAGCAAGCTCAGAGTACAAAAGAGCACTTGGGAAAGAGGAAGTCTTTTCGCCTGCTCAGTGGTCCACG 1300
             ****************************************************************************************************

RNA_SEQ      AGGGTCTGCACAATCACCTTACGACTAAGACCATCTCCCGGTCTCTGGGTAAA                                                1353
BAM          AGGGTCTGCACAATCACCTTACGACTAAGACCATCTCCCGGTCTCTGGGTAAA                                                1353
             *****************************************************
```

(B)

```
RNA_SEQ      CAAATTGTTCTCACCCAGTCTCCAGCAATCATGTCTGCATCTCCAGGGGAGAAGGTCACCATATCCTGCAGTGCCAGCTCAAGTATAAGTTACATGTACT 100
BAM          CAAATTGTTCTCACCCAGTCTCCAGCAATCATGTCTGCATCTCCAGGGGAGAAGGTCACCATATCCTGCAGTGCCAGCTCAAGTATAAGTTACATGTACT 100
             ****************************************************************************************************

RNA_SEQ      GGTACCAGCAGAAGCCAGGATCCTCCCCCAAATCCTGGATTTATCGCACATCCAACCTGGCTTCTGGAGTCCCTGCTCGCTTCAGTGGCAGTGGGTCTGG 200
BAM          GGTACCAGCAGAAGCCAGGATCCTCCCCCAAATCCTGGATTTATCGCACATCCAACCTGGCTTCTGGAGTCCCTGCTCGCTTCAGTGGCAGTGGGTCTGG 200
             ****************************************************************************************************

RNA_SEQ      GACCTCTTACTCTCTCACAATCAGCAGCATGGAGGCTGAAGATGCTGCCACTTATTACTGCCAGCAGTATCATAGTTACCCACGGACGTTCGGTGGAGGC 300
BAM          GACCTCTTACTCTCTCACAATCAGCAGCATGGAGGCTGAAGATGCTGCCACTTATTACTGCCAGCAGTATCATAGTTACCCACGGACGTTCGGTGGAGGC 300
             ****************************************************************************************************

RNA_SEQ      ACCAAGCTGGAAATCAAACGGGCTGATGCTGCACCAACTGTATCCATCTTCCCACCATCCAGTGAGCAGTTAACATCTGGAGGTGCCTCAGTCGTGTGCT 400
BAM          ACCAAGCTGGAAATCAAACGGGCTGATGCTGCACCAACTGTATCCATCTTCCCACCATCCAGTGAGCAGTTAACATCTGGAGGTGCCTCAGTCGTGTGCT 400
             ****************************************************************************************************

RNA_SEQ      TCTTGAACAACTTCTACCCCAAAGACATCAATGTCAAGTGGAAGATTGATGGCAGTGAACGACAAAATGGCGTCCTGAACAGTTGGACTGATCAGGACAG 500
BAM          TCTTGAACAACTTCTACCCCAAAGACATCAATGTCAAGTGGAAGATTGATGGCAGTGAACGACAAAATGGCGTCCTGAACAGTTGGACTGATCAGGACAG 500
             ****************************************************************************************************

RNA_SEQ      CAAAGACAGCACCTACAGCATGAGCAGCACCCTCACGTTGACCAAGGACGAGTATGAACGACATAACAGCTATACCTGTGAGGCCACTCACAAGACATCA 600
BAM          CAAAGACAGCACCTACAGCATGAGCAGCACCCTCACGTTGACCAAGGACGAGTATGAACGACATAACAGCTATACCTGTGAGGCCACTCACAAGACATCA 600
             ****************************************************************************************************

RNA_SEQ      ACTTCACCCATTGTCAAGAGCTTCAACAGGAATGAGTGT                                                              639
BAM          ACTTCACCCATTGTCAAGAGCTTCAACAGGAATGAGTGT                                                              639
             ***************************************
```

Figure S8. Comparison of presented antibody sequencing method (BAM) with results from RNA Illumina sequencing for heavy (A) and light (B) chain.
